# Are Women at Higher Risk for Isolated Surgical Aortic Valve Replacement? Results From 178,000 STS Adult Cardiac Surgery Database Patients

**DOI:** 10.1002/ccd.70188

**Published:** 2025-09-15

**Authors:** Takuya Ogami, Irsa Hasan, Julie A. Phillippi, Derek Serna‐Gallegos, Carlos E. Diaz‐Castrillon, Ibrahim Sultan

**Affiliations:** ^1^ Department of Cardiothoracic Surgery, Division of Cardiac Surgery University of Pittsburgh Pittsburgh Pennsylvania USA; ^2^ Heart and Vascular Institute University of Pittsburgh Medical Center Pittsburgh Pennsylvania USA

**Keywords:** annular enlargement, aortic valve replacement, diversity, gender difference

## Abstract

**Background:**

Female sex is a well‐established risk factor for morbidity and mortality in cardiac surgery.

**Aims:**

This study aimed to assess the characteristics and contemporary outcomes of surgical aortic valve replacement (SAVR) in women compared to men.

**Methods:**

All patients who underwent isolated SAVR from 2014 through 2022 were identified using the Society of Thoracic Surgery national database. Patient characteristics were compared between women and men. The primary interest of outcomes was operative mortality.

**Results:**

A total of 178,014 patients undergoing isolated SAVR were identified, including 64,684 36.3%) women and 113,330 (63.7%) men. Women were older (66.1 years vs. 63.6 years in men, SMD = 0.21) and had a smaller body surface area (1.84 vs. 2.09 m^2^ in men, SMD = 1.15). A history of infective endocarditis was more common in men (10.5% vs. 5.4% in women, SMD = 0.19), while women were more likely to undergo annular enlargement (8.4% vs. 2.9% in men, SMD = 0.24). Propensity score matching yielded 33,228 pairs in each sex category. After matching, operative mortality was comparable (2.2% in women vs. 1.7% in men, SMD = 0.04). Likewise, postoperative complications were similarly observed.

**Conclusion:**

Women undergoing isolated SAVR demonstrated similar morbidity and mortality compared to men despite having smaller body surface area and higher frequency of annular enlargement. Given the improved outcomes with contemporary practice in SAVR, sex may no longer be a risk factor for worse outcomes in isolated SAVR.

## 1 Introduction

Aortic valve disease is commonly seen, with the prevalence of aortic stenosis (AS) ranging from 0.02% to 2.8% and aortic regurgitation (AR) ranging from 0.2% to 2.0%, both increasing with age [1]. In particular, the prevalence of AS rises significantly to 12.4% in individuals aged 75 years or older [2]. In addition to the fact in aortic valve disease, epidemiological and pathophysiological sex differences should be considered.

Historically, data indicated men were twice as likely to develop AS compared to women. More recent data suggest that the sex differences in aortic valve disease might not be so pronounced, but the proportion of hospitalized male patients with AS between 2000 and 2012 was higher at 55.1% than that of female patients based on the Nationwide Inpatient Sample (NIS) Data [3]. Physiological and anatomic differences have historically contributed to different perceptions of risk and the development of aortic valve disease. For instance, men with aortic valve disease tend to be younger than women, likely due to a higher prevalence of bicuspid valve pathology in men, while degenerative disease is more common in women [4, 5]. Women develop severe AS with less calcification than men, and the progression of AS with calcification is faster in men [6, 7]. Additionally, the left ventricular remodeling in response to AS and AR differs between sexes [8, 9]. These observed differences are hypothesized to influence clinical outcomes after surgical aortic valve replacement (SAVR), such as higher morbidity and mortality in women compared to men [10, 11, 12].

However, these studies date back over a decade and findings may be limited by the lack of granularity in surgical data. This study aimed to evaluate contemporary sex‐based outcomes in patients undergoing SAVR using a comprehensive cardiothoracic surgical national database.

## 2 Methods

The Institutional Review Board approval was obtained at University of Pittsburgh Medical Center with the number: STUDY20070004, on 4/22/2021. Informed consent was waived due to the nature of this study.

### 2.1 Data and Patient Selection

The current study was approved by the STS Participant User File Research Program. Deidentified data were obtained from the STS National Adult Cardiac Surgery Database. All patients who underwent isolated SAVR from July 2014 through June 2022 were identified using the database. Patients who underwent transcatheter aortic valve replacement or aortic valve repair were not included. All patients who required coronary artery bypass grafting, other valve surgeries, and aortic surgeries were excluded. The data was collected using data collection form versions 2.81, 2.9, and 4.20.2 during the study period.

### 2.2 Outcomes

The primary outcome of interest was operative mortality, defined as either in‐hospital or 30‐day mortality. The secondary outcomes of interest included cardiopulmonary bypass time, cross‐clamp time, and postoperative complications, including cerebrovascular accident, surgical site infection, sepsis, permanent pacemaker insertion, prolonged ventilation, renal failure, and reoperation for bleeding.

### 2.3 Statistics

Missing data > 5% were excluded from the primary analyses. Continuous variables were compared with *t* test and described as mean ± standard deviations or the Mann−Whitney *U* test and described as median with interquartile range. Normality of distribution was assessed by the Kolmogorov‐Smirnov test. Categorical variables were compared with the Chi‐square test or Fisher's exact test where appropriate. Baseline characteristics and outcomes were compared between women and men. Standardized mean difference (SMD) ≥ 0.1 was considered statistically different. Considering the difference in the baseline characteristics between the sexes, 1:1 nearest neighbor propensity‐score matching (PSM) was applied, using a caliper of 0.2. The selected variables included age, AS, body surface area, creatinine, diabetes, hypertension, peripheral artery disease, liver disease, heart failure, ejection fraction, infective endocarditis, previous myocardial infarction, predicted risk of mortality, cardiopulmonary bypass time, annular enlargement, and urgent or emergent status. After the matching, postoperative outcomes were compared between the matched cohorts. As a sensitivity analysis, postoperative outcomes were compared in patients who underwent elective SAVR, excluding urgent or emergent cases and patients with a history of infective endocarditis. The second sensitivity analysis was performed excluding patients who underwent annular enlargement. The third sensitivity analysis was conducted in subgroups based on age categories (age ≤ 50, 50 < age ≤ 75, 75 < age). Bar graphs were drawn to describe the annual trend of prosthetic valve use. All statistical analysis was performed using R version 4.1.3 (R Foundation for Statistical Computing, Vienna, Austria).

## 3 Results

A total of 178,014 patients who underwent isolated SAVR were identified: 64,684 (36.3%) women and 113,330 (63.7%) men. While the case volume of SAVR has been stable in age of 50 or younger, it has declined in patients older than 70 years old over the period (Supplemental Figure 1). AS was present in 82.4% (*n* = 143,732) and more than moderate or severe AR was present in 42.4% (*n* = 68,486). Resternotomy was performed in 11.5% (*n* = 20,533), including redo intervention in aortic valve position in 8.4% (*n* = 14,865). Annular enlargement was performed in 8,701 (4.9%). Mechanical valve was used in 13.5% (*n* = 23,659). Operative mortality was 1.9%.

### 3.1 Sex Differences Before PSM

Women were older (66.1 vs. 63.6 years in men, SMD = 0.21) and had smaller body surface area (1.84 vs. 2.09 m^2^ in men, SMD = 1.15) (Table 1). A history of infective endocarditis was more common in men (10.5% vs. 5.4% in women, SMD = 0.19). AS was more common in women (88.5% vs. 78.9% in men, SMD = 0.26). Women were more likely to undergo annular enlargement (8.4% vs. 2.9% in men, SMD = 0.24) (Table 2). Despite the baseline differences between the two cohorts, unadjusted risk of operative mortality was similar (2.3% in women vs. 1.6% in men, SMD = 0.05) (Table 3).

**Table 1:** Baseline characteristics.

	Before PSM			After PSM		
Variables (%)	Women *N* = 64,684	Men *N* = 113,330	SMD	Women *N* = 33,228	Men *N* = 33,228	SMD
Age (mean ± SD, years)	66.1 ± 11.3	63.6 ± 12.3	0.21	64.8 ± 10.9	65.8 ± 13.1	0.08
Body surface area (mean ± SD, kg/m^2^)	1.84 ± 0.22	2.09 ± 0.22	1.15	1.95 ± 0.20	1.94 ± 0.19	0.02
White race	55,223 (87.8)	96,899 (88.1)	0.01	28,708 (88.3)	28,098 (86.9)	0.04
Non‐White race	7686 (12.2)	13,076 (11.9)	0.01	3819 (11.7)	4254 (13.1)	0.04
Comorbidities						
Hypertension	50,377 (78.0)	87,833 (77.6)	0.01	25,858 (77.8)	26,028 (78.3)	0.01
Diabetes mellitus	5835 (14.2)	2981 (20.0)	0.16	10,106 (30.4)	10,368 (31.2)	0.02
Dialysis	1090 (1.7)	2575 (2.3)	0.04	682 (2.1)	378 (1.1)	0.07
Previous MI	4919 (7.6)	12,874 (11.4)	0.13	3054 (9.2)	3055 (9.2)	< 0.001
Liver disease	2045 (3.2)	5248 (4.7)	0.08	1288 (3.9)	1256 (3.8)	0.01
Peripheral artery disease	4287 (6.7)	8704 (7.7)	0.04	2323 (7.0)	2496 (7.5)	0.02
Chronic lung disease	15,384 (24.2)	25,400 (22.8)	0.03	24,762 (75.5)	25,096 (76.6)	0.03
Cerebrovascular disease	10,366 (16.1)	16,776 (14.9)	0.03	5354 (16.2)	5175 (15.6)	0.02
History of heart failure	24,103 (38.1)	42,899 (38.7)	0.01	12,580 (37.9)	11,856 (35.7)	0.05
Permanent pacemaker	1542 (2.4)	3309 (2.9)	0.03	769 (2.3)	1029 (3.1)	0.05
Ejection fraction	59.7 ± 9.57	56.2 ± 11.3	0.3	58.5 ± 9.90	58.5 ± 10.6	0.001
Aortic stenosis	56,220 (88.5)	87,512 (78.9)	0.26	28,454 (85.6)	29223 (87.9)	0.07
Aortic regurgitation						
None	11,663 (20.2)	14,815 (14.3)		6009 (20.2)	5453 (17.4)	
Trace	10,052 (17.4)	14,680 (14.2)		4990 (16.7)	4778 (15.3)	
Mild	15,864 (27.4)	25,879 (25.0)		8070 (27.1)	8791 (28.1)	
Moderate	9406 (16.3)	17783 (17.2)		4715 (15.8)	5778 (18.5)	
Severe	10,831 (18.7)	30,466 (29.4)		6026 (20.2)	6517 (20.8)	
Infective endocarditis	3473 (5.4)	11,932 (10.5)	0.19	2333 (7.0)	2022 (6.1)	0.04
Urgent or Emergent	11,059 (17.1)	24,576 (21.7)	0.12	6360 (19.1)	6110 (18.4)	0.02
Previous cardiac surgery	6489 (10.0)	14044 (12.4)	0.08	3432 (10.3)	3576 (10.8)	0.01
Aortic position	5228 (8.1)	9637 (8.5)	0.02	2456 (7.0)	2714 (7.7)	0.03
Explant TAVR	337 (0.5)	664 (0.6)	0.01	184 (0.6)	126 (0.4)	0.03
STS PROM (mean ± SD, %)	2.60 ± 3.07	1.86 ± 2.69	0.26	2.34 ± 2.74	2.36 ± 3.84	0.004

Abbreviations: MI, myocardial infarction; PSM, propensity‐score matching; SMD, standardized mean difference; STS PROM, Society of Thoracic Surgery predicted risk of mortality; TAVR, transcatheter aortic valve replacement.

**Table 2:** Operative data.

	Before PSM			After PSM		
Variables (%)	Women *N* = 64,684	Men *N* = 113,330	SMD	Women *N* = 33,228	Men *N* = 33,228	SMD
Mechanical valve	7956 (12.5)	15703 (14.1)	0.05	4540 (13.8)	3736 (11.3)	0.08
Annular enlargement	5393 (8.4)	3308 (2.9)	0.24	1756 (5.3)	1658 (5.0)	0.01
CPB time (median [IQR], min)	96.0 [77.0, 121.0]	90.0 [72.0, 114.0]	0.14	92.0 [73.0, 116.0]	93.0 [74.0, 116.0]	< 0.001
Cross clamp time (median [IQR], min)	72.0 [57.0, 90.0]	68.0 [54.0, 86.0]	0.13	69.0 [55.0, 87.0]	70.0 [56.0, 87.0]	0.01

Abbreviations: CPB, cardiopulmonary bypass; PSM, propensity‐score matching; SMD, standardized mean difference.

**Table 3:** Postoperative outcomes.

	Before PSM			After PSM		
Variables (%)	Women *N* = 64,684	Men *N* = 113,330	SMD	Women *N* = 33,228	Men *N* = 33,228	SMD
Operative mortality	1512 (2.3)	1843 (1.6)	0.05	719 (2.2)	557 (1.7)	0.04
Cerebrovascular accident	853 (1.3)	1224 (1.1)	0.02	390 (1.2)	384 (1.2)	0.002
Surgical site infection	95 (0.1)	168 (0.1)	< 0.001	55 (0.2)	48 (0.1)	0.01
Sepsis	473 (0.7)	858 (0.8)	0.003	215 (0.6)	283 (0.9)	0.02
Permanent pacemaker insertion	3337 (5.2)	4951 (4.4)	0.04	1603 (4.8)	1459 (4.4)	0.02
Prolonged ventilation	4264 (6.6)	6756 (6.0)	0.03	2108 (6.3)	1968 (5.9)	0.02
Renal failure	1096 (1.7)	1995 (1.8)	0.01	545 (1.6)	555 (1.7)	0.002
Reoperation for bleeding	1474 (2.3)	3615 (3.2)	0.06	628 (1.9)	1112 (3.3)	0.09
Length of stays (median [IQR], days)	6.0 [5.0, 8.0]	5.0 [4.0, 7.0]	0.04	6.0 [5.0, 8.0]	5.0 [4.0, 7.0]	0.05

Abbreviations: PSM, propensity‐score matching; SMD, standardized mean difference.

### 3.2 Characteristics After PSM

**Figure 1:**
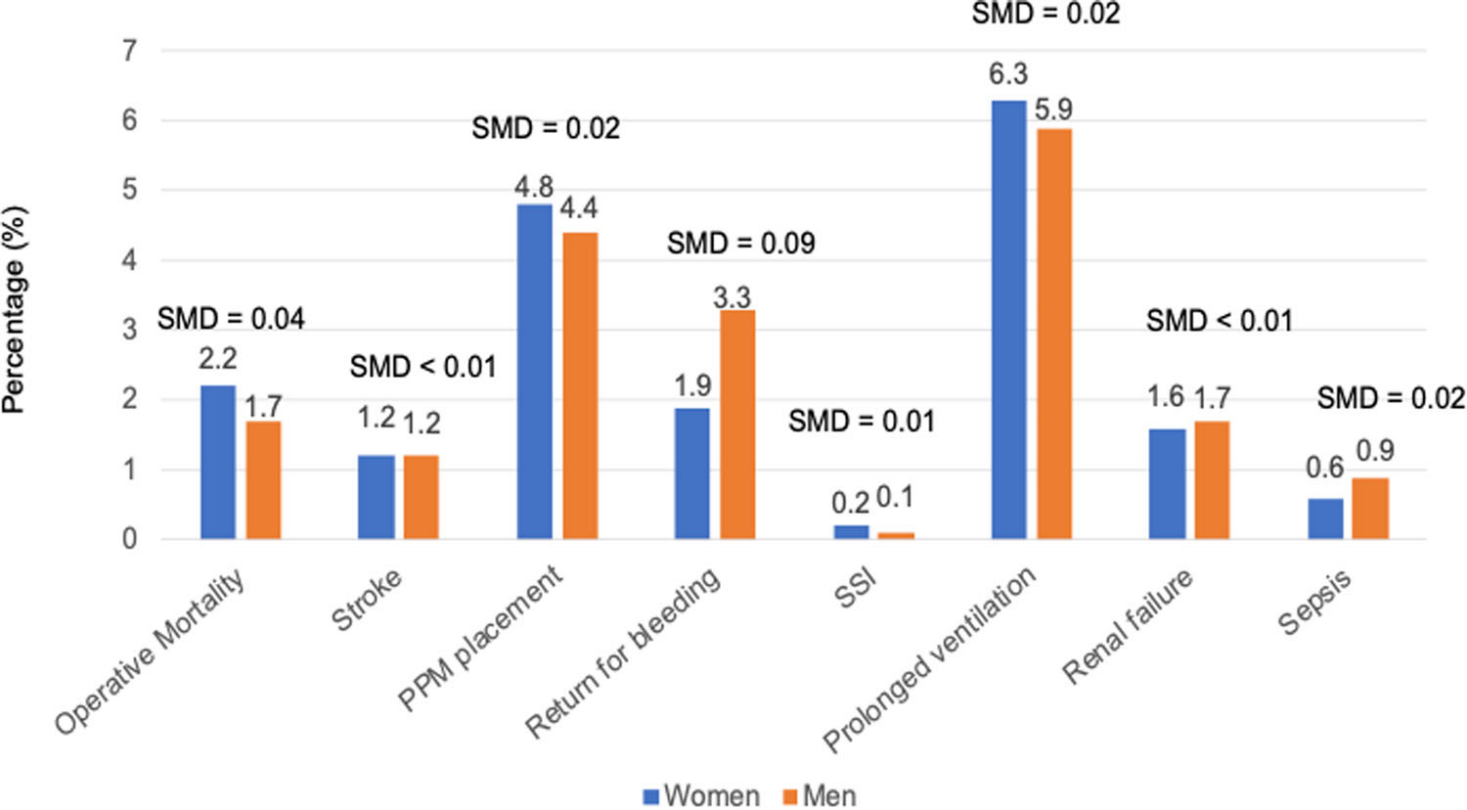
Outcomes after aortic valve replacement between women and men after matching. The number on each bar graph represents frequency of complications. Outcomes were compared between women and men after propensity‐score matching. SMD > 0.1 was considered statistically different. PPM, permanent pacemaker; SMD, standardized mean difference; SSI, surgical site infection. [Color figure can be viewed at wileyonlinelibrary.com]

Propensity score matching yielded 33,228 pairs in each sex (Table 1). Annular enlargement was performed in 5.3% of women versus 5.0% of men (SMD = 0.09, Table 2). Mechanical valve use was similar between women and men (13.8% vs. 11.3%, respectively, SMD = 0.08). The mean cardiopulmonary bypass time was equivalent (99.0 min in both sexes, SMD < 0.001). Operative mortality was comparable between sexes (2.2% in women vs. 1.7% in men, SMD = 0.04) (Table 3, Figure 1). Stroke rate was 1.2% in both sexes (SMD = 0.002). New permanent pacemaker insertion was performed equally between women and men (4.8% vs. 4.4%, respectively, SMD = 0.02). Reoperation for bleeding was 1.9% in women and 3.3% in men (SMD = 0.09). Likewise, the rest of the complications were similarly observed between the groups.

### 3.3 Sensitivity Analyses

In a subgroup of patients who underwent elective SAVR, excluding patients with a history of infective endocarditis, postoperative outcomes remained similar between women and men (Table S1). Other subgroup analyses excluding patients who underwent annular enlargement and with age categories demonstrated consistent findings (Tables S2 and S3).

### 3.4 Trend in Valve Choice

**Figure 2:**
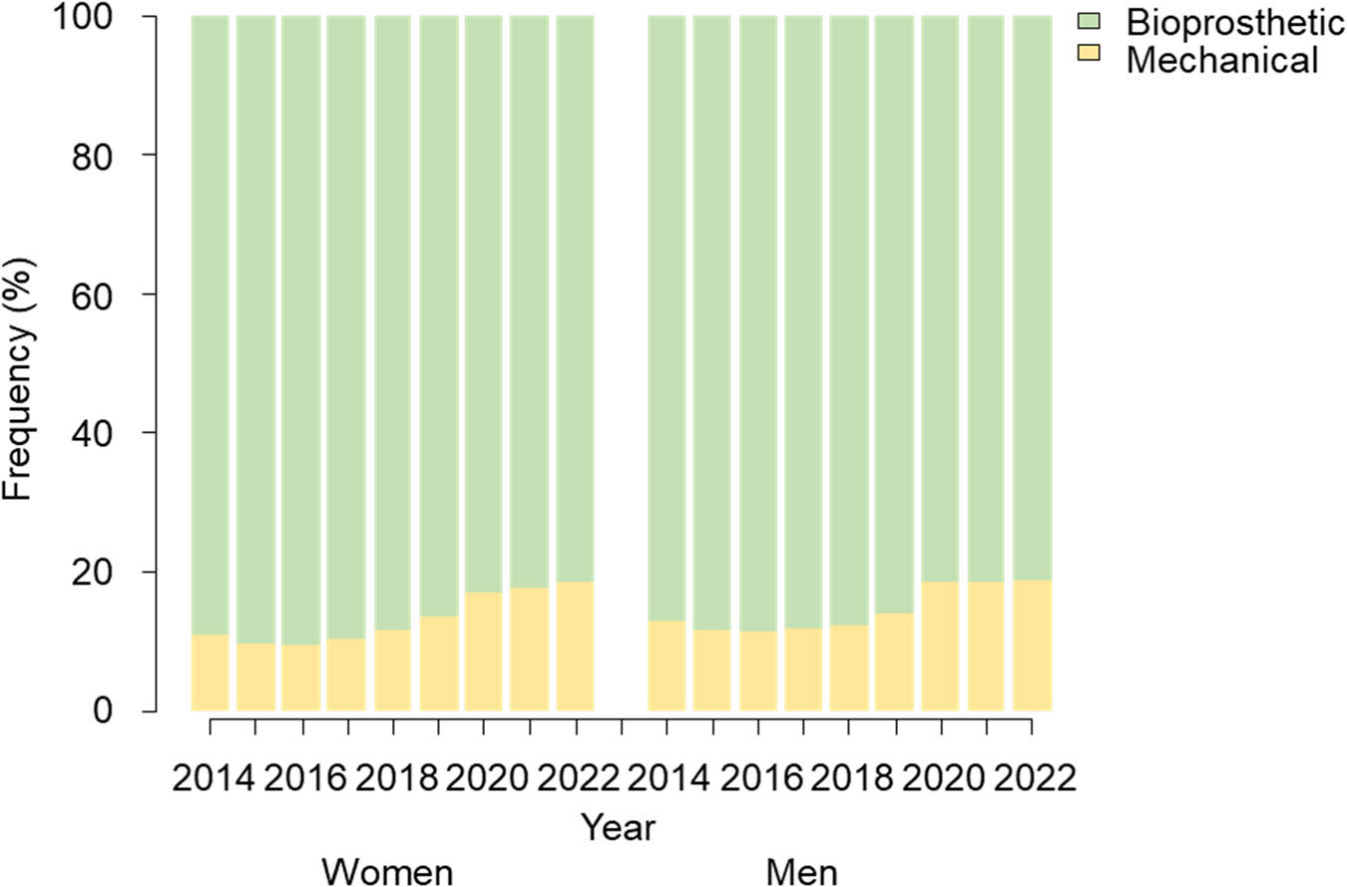
The trend of the mechanical valve to bioprosthetic valve ratio between women and men. Mechanical valve to bioprosthetic valve ratio was plotted against year of surgery in women and men. [Color figure can be viewed at wileyonlinelibrary.com]

The number of mechanical valve utilization to the prosthetic valve has slowly increased from 10.9% in 2014 to 18.6% in 2022 in women who underwent SAVR (Figure 2). Likewise, the absolute number of mechanical valve use was up trending from 795 in 2020 to 1078 in 2022 while the numbers trended down between 2015 and 2019 (Figure S2). Similar trends were observed in men. However, the use of mechanical valve in women younger than 50 years old, which was considered childbearing age, was constant over the period (Figure S3).

## 4 Discussion

The present study studied sex differences among 178,014 patients undergoing isolated SAVR from 2014 to 2022 using the STS national database, encompassing the most comprehensive cardiothoracic surgery data available. The main findings of this study are as follows: (1) morbidity and mortality were comparable between women and men and persisted after risk adjustment, (2) women had smaller body surface area and more frequently underwent annular enlargement than men, and (3) the proportion of mechanical valve use increased in women over time. However, it was not as obvious in those aged less than 50 years.

Overall operative mortality in women undergoing isolated SAVR was 2.3% in the present study. This cohort included cases with higher operative risk such as infective endocarditis, redo sternotomy, and annular enlargement. Historically, women were believed to have a higher risk of morbidity and mortality in cardiac surgery, as reflected in the STS predicted risk of mortality and EuroSCORE [13, 14]. However, this study found mortality was equivalent between women and men using PSM in the contemporary era. A potential explanation for the discrepancy between the current study and the historical data may be attributed to the overall improved management and outcomes in cardiac surgery, cardiac anesthesia, and perioperative critical care. A retrospective study using the NIS database found a 3.3% in‐hospital mortality in women undergoing isolated SAVR but found that there was a decreasing trend in mortality over the study period from 2003 to 2014. Additionally, contemporary data support that sex differences in mortality may be becoming more subtle as SAVR outcomes have improved. Using a systematic review, the authors found women had similar in‐hospital or 30‐day mortality to men following SAVR [15]. Additional consideration for conflicting results between contemporary and historical studies should focus on statistical methodology. The difference in mortality between sexes was 0.5% after PSM in this study (2.2% in women and 1.7% in men), suggesting no difference in mortality between sexes both statistically and clinically.

Even though mortality outcomes may not differ, intraoperative challenges of SAVR performed in women still exist. In this study, women had a smaller body surface area and more often required annular enlargement compared to men, which aligns with previously published data [16, 17, 18]. This is unsurprising considering smaller body and annulus sizes in females, leading to a greater need for annular enlargement. Placing a smaller valve may complicate future valve‐in‐valve procedures when selecting a bioprosthetic valve, and surgeons should anticipate a potential challenge when operating on women with smaller body surface areas, necessitating annular enlargement.

In a previous STS study, the absolute number of mechanical valve utilization was overall declining among SAVR patients [19]. However, the current study demonstrates that the proportion of mechanical valve use increased in the past decade in both sexes. This finding is likely a consequence of patients who undergo bioprosthetic valve replacement are increasingly undergoing TAVR [20, 21, 22]. Particularly, the decline in annual SAVR cases among patients older than 70 years old may support this speculation. The growing absolute number of mechanical valves used in the past couple of years in both sexes may also reflect the potential survival benefit of mechanical valves and guidelines recommendations in the younger population [23, 24]. For example, American College of Cardiology/American Heart Association guidelines recommend mechanical valves specifically for those less than 50 years old [22]. Moreover, the emergence of newer‐generation valves may have facilitated the use of mechanical valves [25, 26]. Newer‐generation mechanical valves allow for a lower international normalized ratio target after valve implantation, potentially reducing bleeding risk. Consequently, they may be preferred over future valve‐in‐valve options after bioprosthetic valve implantation, especially in young patients or patients with a small annulus. Notably, the increasing use of mechanical valves over bioprosthetic valves was not specifically observed in women of childbearing age in the current study, where the requirement for anticoagulation with mechanical valves complicates pregnancy and delivery [22]. While it is speculated that women planning to have children were more likely to receive bioprosthetic valves during SAVR for this reason, it cannot be concluded from the current study, as pregnancy‐related data were unavailable.

The present study has several limitations, including inherent limitations present in an administrative database study. First, long‐term mortality was unavailable in this study while conflicting results have been reported in terms of sex differences in long‐term mortality after SAVR; therefore, conclusions cannot be reliably derived for long‐term outcomes [15, 27]. However, the lack of significant sex differences in immediate postoperative data following SAVR is a novel insight. Second, selection bias cannot be excluded. For example, some women might have been precluded from SAVR due to higher predicted risks of morbidity and mortality. Female patients involved in the current study might be lower‐risk surgical candidates. Third, pregnancy and delivery‐related data were unavailable, which might have an impact on outcomes in the younger female population.

In conclusion, the present study demonstrated comparable outcomes between sexes in patients who underwent isolated SAVR. Women were smaller and underwent annular enlargement more often than men, leading to speculation that they might experience higher morbidity and mortality. However, this observation did not impact clinical outcomes between sexes, even before PSM. Female sex may no longer be a risk factor for worse outcomes in isolated SAVR in the contemporary era.

## Conflicts of Interest

Dr. Sultan receives institutional research support from Abbott, Atricure, Artivion, WL Gore, Edwards, Medtronic, and Terumo Aortic. The other authors declare no conflicts of interest.

## Supporting information


**Supplemental Figure 1:** The trend of surgical aortic valve replacement volume per year in age categories Legend: The number on each bar graph represents case numbers per year stratified by age categories. **Supplemental Figure 2:** The trend of the absolute number of mechanical valve use between sexes Legend: The number on each bar graph represents case numbers per year. **Supplemental Figure 3:** The trend of the mechanical valve to bioprosthetic valve ratio in women stratified by age.

**Supplemental Table 1:** Postoperative outcomes after PSM in elective case. **Supplemental Table 2:** Postoperative outcomes after PSM excluding patients who underwent annular enlargement. **Supplemental Table 3:** Postoperative outcomes stratified by age category after PSM.
