# Supplementary material for: Are Women at Higher Risk for Isolated Surgical Aortic Valve Replacement? Results From 178,000 STS Adult Cardiac Surgery Database Patients
Source: Catheter Cardiovasc Interv. 2025 Sep 15;106(6):3223–9. doi: 10.1002/ccd.70188 (PMC12617330; doi:10.1002/ccd.70188)
Supplement: Supplementary file 1 — Supplemental Figure 1: The trend of surgical aortic valve replacement volume per year in age categories Legend: The number on each bar graph represents case numbers per year stratified by age categories. Supplemental Figure 2: The trend of the absolute number of mechanical valve use between sexes Legend: The number on each bar graph represents case numbers per year. Supplemental Figure 3: The trend of the mechanical valve to bioprosthetic valve ratio in women stratified by age. [file CCD-106-3223-s002.docx]

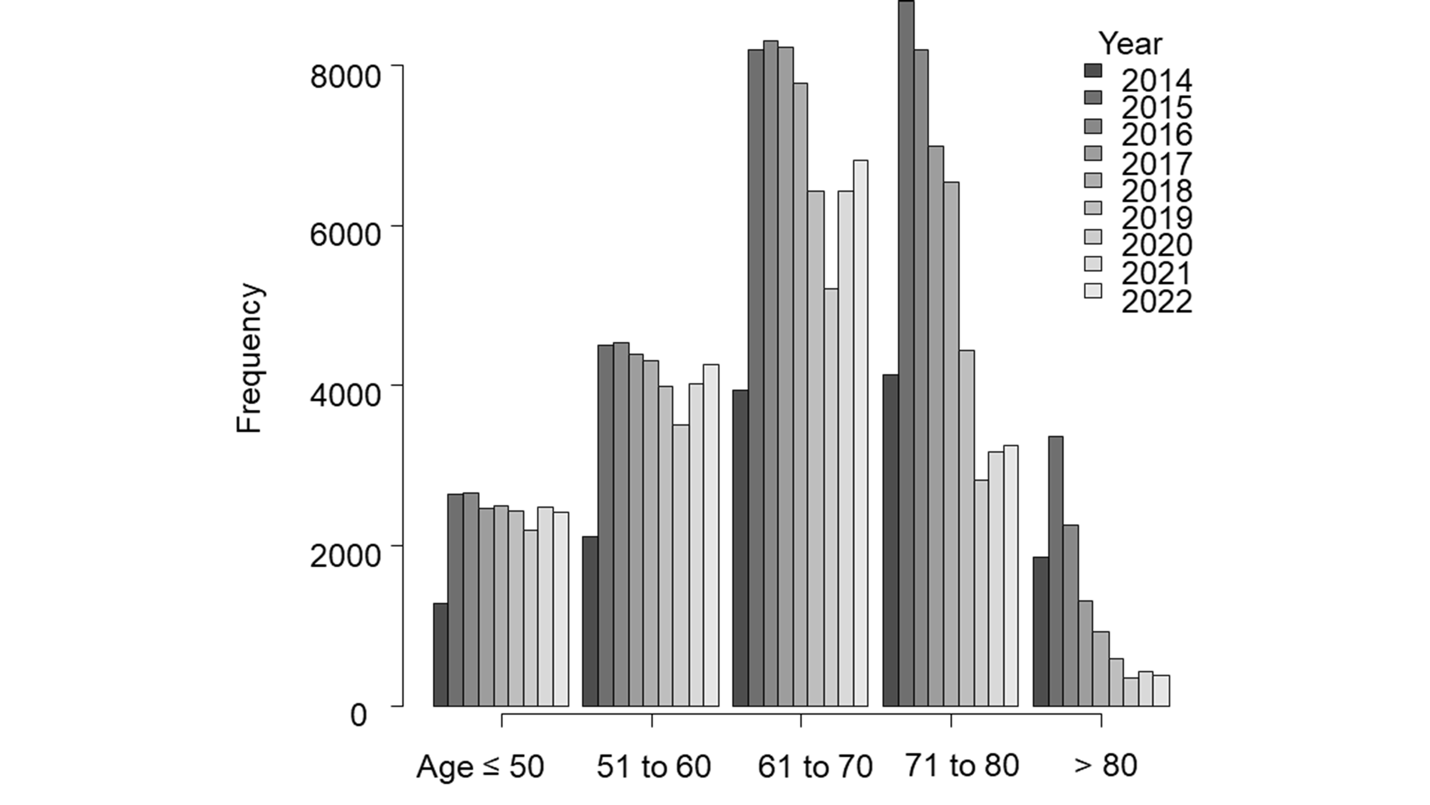


**Supplemental Figure 1** The trend of surgical aortic valve replacement volume per year in age categories

Legend: The number on each bar graph represents case numbers per year stratified by age categories.


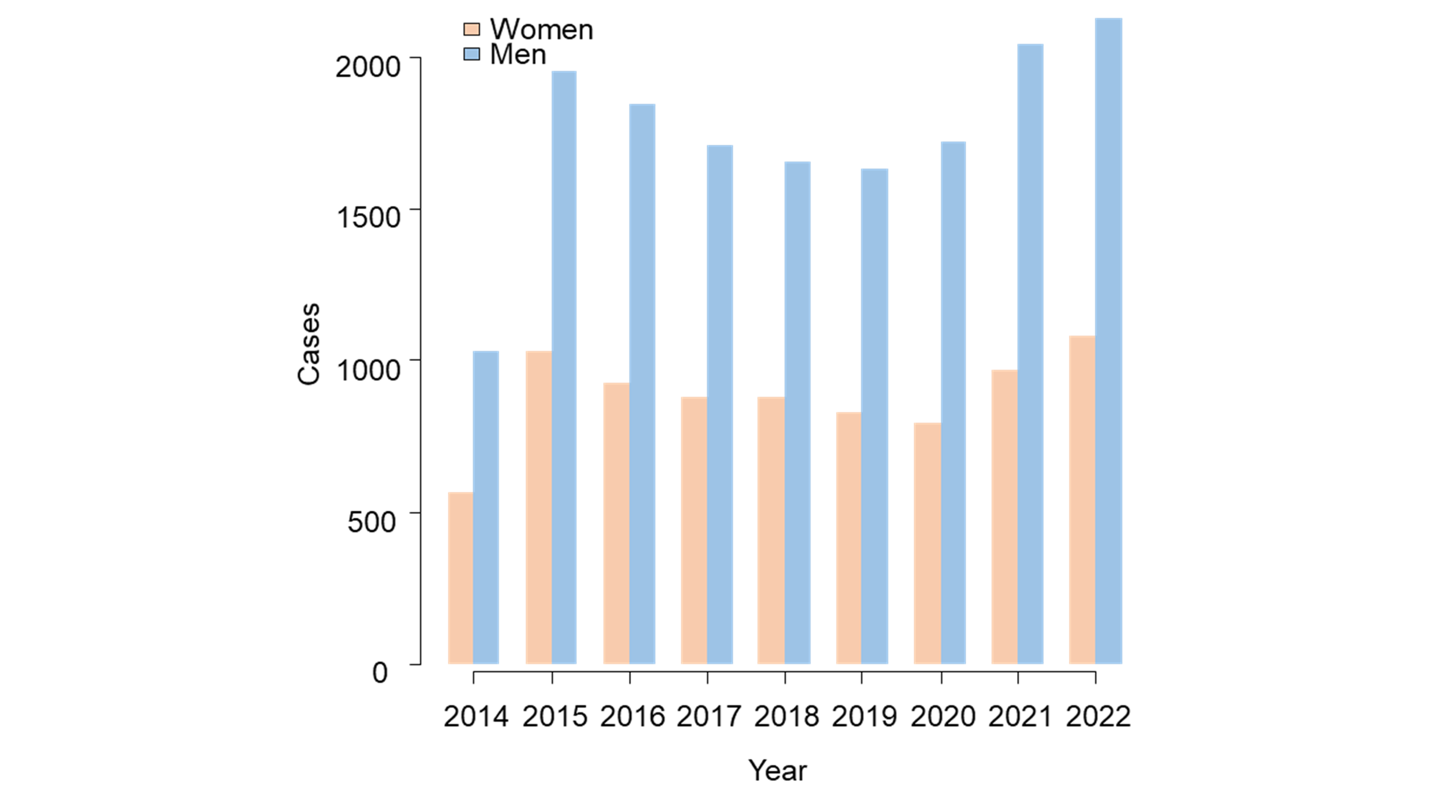


**Supplemental Figure 2** The trend of the absolute number of mechanical valve use between sexes

Legend: The number on each bar graph represents case numbers per year.


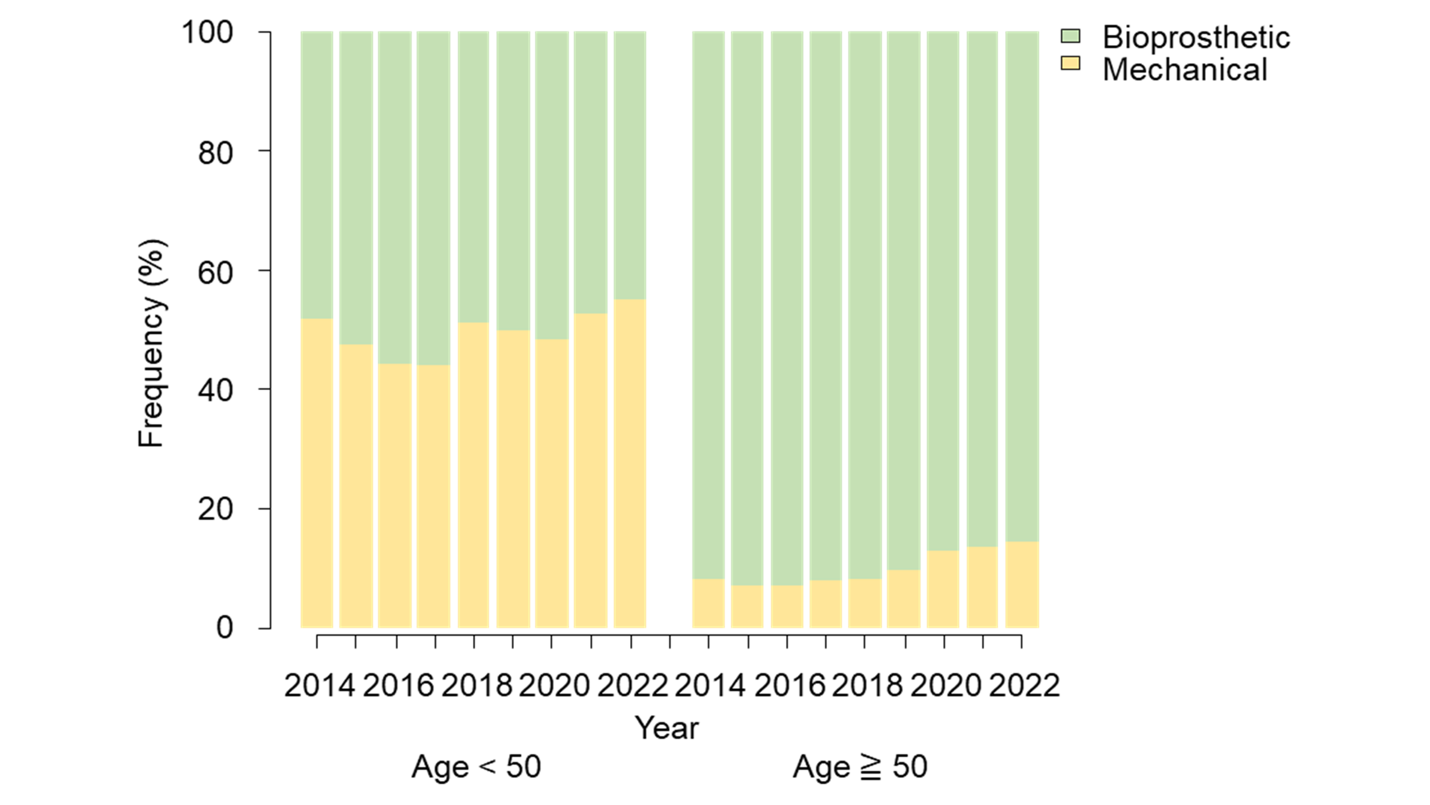


**Supplemental Figure 3** The trend of the mechanical valve to bioprosthetic valve ratio in women stratified by age

Legend: Mechanical valve to bioprosthetic valve ratio was plotted against year of surgery in women stratified by age of 50 which may be used to define childbearing age.
