# Supplementary material for: Are Women at Higher Risk for Isolated Surgical Aortic Valve Replacement? Results From 178,000 STS Adult Cardiac Surgery Database Patients
Source: Catheter Cardiovasc Interv. 2025 Sep 15;106(6):3223–9. doi: 10.1002/ccd.70188 (PMC12617330; doi:10.1002/ccd.70188)
Supplement: Supplementary file 2 — Supplemental Table 1: Postoperative outcomes after PSM in elective case. Supplemental Table 2: Postoperative outcomes after PSM excluding patients who underwent annular enlargement. Supplemental Table 3: Postoperative outcomes stratified by age category after PSM. [file CCD-106-3223-s001.docx]

| Supplemental Table 1. Postoperative outcomes after PSM in elective case | | | |
| --- | --- | --- | --- |
| Variables (%) | Women  N=22,564 | Men  N=22,564 | SMD |
| Operative mortality | 345 (1.5) | 271 (1.2) | 0.03 |
| Cerebrovascular accident | 215 (1.0) | 239 (1.1) | 0.01 |
| Surgical site infection | 35 (0.2) | 39 (0.2) | 0.004 |
| Sepsis | 91 (0.4) | 151 (0.7) | 0.04 |
| Permanent pacemaker insertion | 1040 (4.6) | 899 (4.0) | 0.03 |
| Prolonged ventilation | 977 (4.3) | 954 (4.2) | 0.01 |
| Renal failure | 273 (1.2) | 275 (1.2) | 0.001 |
| Reoperation for bleeding | 381 (1.7) | 710 (3.1) | 0.09 |
| Length of stays (median [IQR], days) | 6.00 [5.00, 7.00] | 5.00 [4.00, 7.00] | 0.03 |
| PSM, propensity-score matching; SMD, standardized mean difference | | | |

| Supplemental Table 2. Postoperative outcomes after PSM excluding patients who underwent annular enlargement | | | |
| --- | --- | --- | --- |
| Variables (%) | Women  N=31,655 | Men  N=31,655 | SMD |
| Operative mortality | 630 (2.0) | 538 (1.7) | 0.02 |
| Cerebrovascular accident | 364 (1.1) | 366 (1.2) | 0.001 |
| Surgical site infection | 51 (0.2) | 47 (0.1) | 0.003 |
| Sepsis | 187 (0.6) | 273 (0.9) | 0.03 |
| Permanent pacemaker insertion | 1515 (4.8) | 1407 (4.4) | 0.02 |
| Prolonged ventilation | 1902 (6.0) | 1922 (6.1) | 0.003 |
| Renal failure | 479 (1.5) | 536 (1.7) | 0.01 |
| Reoperation for bleeding | 583 (1.8) | 1088 (3.4) | 0.1 |
| Length of stays (median [IQR], days) | 6.00 [5.00, 8.00] | 5.00 [4.00, 7.00] | 0.04 |
| PSM, propensity-score matching; SMD, standardized mean difference | | | |

| Supplemental Table 3. Postoperative outcomes stratified by age category after PSM | | | | | | | | | |
| --- | --- | --- | --- | --- | --- | --- | --- | --- | --- |
|  | Age ≤ 50 | | | 50 < Age ≤ 75 | | | 75 < Age | | |
| Variables (%) | Women  N=3,828 | Men  N=3,828 | SMD | Women  N=23,092 | Men  N=23,092 | SMD | Women  N=5,682 | Men  N=5,682 | SMD |
| Operative mortality | 70 (1.8) | 60 (1.6) | 0.02 | 466 (2.0) | 308 (1.3) | 0.05 | 177 (3.1) | 164 (2.9) | 0.01 |
| Cerebrovascular accident | 31 (0.8) | 48 (1.3) | 0.04 | 239 (1.0) | 224 (1.0) | 0.01 | 100 (1.8) | 96 (1.7) | 0.01 |
| Surgical site infection | 11 (0.3) | 5 (0.1) | 0.04 | 39 (0.2) | 33 (0.1) | 0.01 | 10 (0.2) | 10 (0.2) | <0.001 |
| Sepsis | 31 (0.8) | 31 (0.8) | <0.001 | 145 (0.6) | 166 (0.7) | 0.01 | 38 (0.7) | 85 (1.5) | 0.08 |
| Permanent pacemaker insertion | 174 (4.5) | 132 (3.5) | 0.06 | 1092 (4.7) | 952 (4.1) | 0.03 | 342 (6.0) | 329 (5.8) | 0.01 |
| Prolonged ventilation | 302 (7.9) | 267 (7.0) | 0.04 | 1374 (6.0) | 1279 (5.5) | 0.02 | 421 (7.4) | 423 (7.4) | 0.001 |
| Renal failure | 63 (1.6) | 75 (2.0) | 0.02 | 331 (1.4) | 322 (1.4) | 0.003 | 145 (2.6) | 136 (2.4) | 0.01 |
| Reoperation for bleeding | 63 (1.6) | 128 (3.3) | 0.11 | 430 (1.9) | 722 (3.1) | 0.08 | 126 (2.2) | 232 (4.1) | 0.11 |
| Length of stays (median [IQR], days) | 6.00 [5.00, 8.00] | 5.00 [4.00, 8.00] | 0.08 | 6.00 [5.00, 7.00] | 5.00 [4.00, 7.00] | 0.07 | 6.00 [5.00, 8.00] | 6.00 [5.00, 8.00] | 0.05 |
| PSM, propensity-score matching; SMD, standardized mean difference | | | | | | | | | |
